# Mine Vegetation Identification via Ecological Monitoring and Deep Belief Network

**DOI:** 10.3390/plants10061099

**Published:** 2021-05-30

**Authors:** Bin Gong, Cheng Shu, Song Han, Sheng-Gao Cheng

**Affiliations:** School of Environmental Studies, China University of Geosciences, Wuhan 430074, China; gongbin1983@126.com (B.G.); cughan2012@126.com (S.H.); chengsg@cug.edu.cn (S.-G.C.)

**Keywords:** remote sensing image, deep learning, deep belief network, mine, vegetation, ecological monitoring

## Abstract

Based on the characteristics of remote sensing images of mine vegetation, this research studied the application of deep belief network model in mine vegetation identification. Through vegetation identification and classification, the ecological environment index of mining area was determined according to the analysis of vegetation and coverage. Deep learning algorithm is adopted to improve the depth study, the vegetation coverage in the analysis was studied. Parameters and parameter values were selected for identification by establishing the optimal experimental design. The experimental results were compared with remote sensing images to determine the accuracy of deep learning identification and the effectiveness of the algorithm. When the sample size is 2,000,000 pixels, through repeated tests and classification effect comparison, the optimal parameter setting suitable for mine vegetation identification is obtained. Parameter setting: the number of network layers is 3 layers; the number of hidden layer neurons is 60. The learning rate is 0.01 and the number of iterations is 2. The average recognition rate of vegetation coverage was 95.95%, outperforming some other models, and the accuracy rate of kappa coefficient was 0.95, which can accurately reflect the vegetation coverage. The clearer the satellite image is, the more accurate the recognition result is, and the accuracy is closer to 100%. The identification of vegetation coverage has important guiding significance for determining the area and area of ecological restoration.

## 1. Introduction

With the increasing intensity of mineral resources development, particularly the long-term, poorly planned, and unreasonable development, mining has resulted in quite serious environmental problems. It affects the living environment and economic development of regional residents, thus bringing about potential social problems [1]. The environmental quality of a mine was analyzed and assessed by scientific and efficient means, and effective protection and prevention measures for the mine environment were implemented to achieve sustainable development [2]. Remote sensing image recognition and classification has been among the hotspots in the field of image processing during recent years [3].

Remote sensing technology is widely used in military research [4], land-use change monitoring [5], environmental monitoring [6], and geological geography [7]. During the late 1990s, the MINE project used Earth observation technology to monitor the impact of mining activities on the environment. The use of hyperspectral and multispectral remote sensing imagery to monitor the mine environment under different climates and mines types yielded fruitful results [8]. In 2010, E. Charou and others monitored [9] the surface change and water body of a mine by using multi-temporal remote sensing data of different resolutions and demonstrated the feasibility of remote sensing combined with Geographic Information System technology for long-term monitoring of a mine environment. Z. Chunlan and others [10] used the technology of remote sensing and geo-information to monitor the ecological environment of the Panzhihua mine and analyzed the area change in vegetation cover type caused by mining in the area. With the successive launch of China’s remote sensing satellites, the quantity and quality of domestic remote sensing data have been greatly improved. Remote sensing data of different resolutions have also been applied to mine environmental monitoring. L. Yunge and others [11] completed remote sensing monitoring of 1:10,000 scale mine in the key mining area of Mozhugongka in Tibet by using “Gaofen-2 satellite” remote sensing data.

The method of extracting remote sensing information continues to face great challenges in practical applications; however, an image recognition method based on machine learning provides the possibility of solving this problem. Deep learning is the emerging direction of machine learning research during recent years [12]. The main working principle of Alpha Go, which has been recently popular, is deep learning. Deep learning originates from a neural network, which mimics the basic structure of the neurons in the human brain, and extracts data from the lower to upper layers. It can improve recognition accuracy by discovering the characteristics of the data in time and space [13]. Convolutional neural networks [14], deep belief networks [15], and stack-type automatic encoders are several common models in deep learning. The deep belief network (DBN) was proposed by Professor Hinton in the University of Toronto and his students in 2006, which triggered a wave of deep learning. DBN is a deep learning model that combines the advantages of supervised and unsupervised classification [16]. It consists of several restricted Boltzmann machines (RBMs) that can classify and reduce the dimensions of data and maximize the use of deep networks to fit linear and nonlinear classification problems to reduce classification errors [17]. In 2016, Guanyu C. [18] applied a convolutional neural network method in a deep learning algorithm to identify and classify bad geological bodies. Further, the classification accuracies were compared by using the K-means, support vector machine, and Bayesian classifiers. When there are sufficient training samples, the classification results of the deep learning algorithm will exhibit obvious advantages. Compared with the shallow machine model, the deep learning model can realize the approximation representation of complex functions with fewer parameters, thus learning the essential data characteristics. Based on the DBN algorithm model, a remote sensing image of a mine area was used to analyze and classify the vegetation and cover and the ecological environment index of the mine via the vegetation and coverage rate to monitor mine ecological development.

In this research, the deep belief network was used to identify and analyze the vegetation coverage in the study area. The parameters were selected through experimental design and the optimal parameter values were set. The experimental results were compared with remote sensing images to determine the accuracy and effectiveness of the deep learning algorithm identification. The identification results can accurately reflect the vegetation covered areas and non-vegetation covered areas, and effectively determine the scope and area of ecological vegetation restoration. It is of great significance to the development of ecological environment monitoring technology and provides data support for territorial spatial environment planning and decision-making departments..

## 2. Research Areas and Methods

### 2.1. General Situation of The Study AreaF

The study area is Maweishan City Forest Park in Huangmei County, Huanggang City, Hubei Province. Mawei Mountain is an ecological barrier in Huangmei County, which is rich in iron ore, clay, and other resources. Since the 1980s, because of the lack of management, limited awareness of environmental protection, poorly planned mining, and simple mining technology mostly based on open-pit mining, no effective ecological protection and restoration measures have been implemented in the mining process. This has resulted in severe damage of the ecological environment in Maweishan including removal of a large amount of vegetation and deterioration of the mountain forest ecosystem.

**Figure 1:**
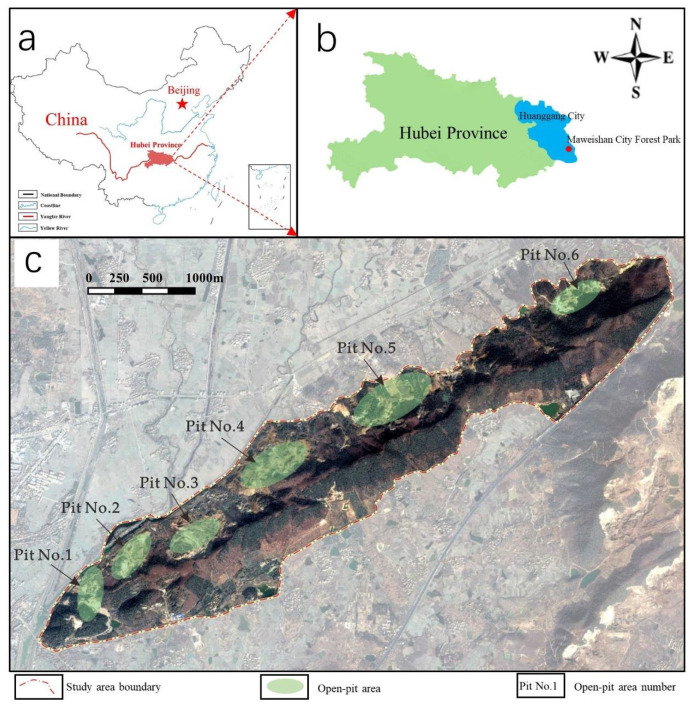
Overall location map and distribution map of Mawei Mountain iron ore pits. (**a**) The position of Hubei Province in China. (**b**) The location of Maweishan City Forest Park in Hubei Province. (**c**) Research Areas of Mawei Mountain iron ore pits.

The present situation of abandoned land on Mawei Mountain is shown in Figure 1. The mining of iron ore is mainly concentrated on the northern part of Mawei Mountain. From the existing information of the study area, it is evident that there are many large or small mining faces on the north side of Mawei Mountain. According to the damage degree of mining faces shown by the satellite image, the entire Maweishan mining area was divided into six main open-pit areas. Pit Nos. 1, 2, 3, and 4 are the most representative and also the most seriously damaged areas. They are the focus of ecological restoration and landscape reconstruction during the development of the Maweishan City Forest Park.

### 2.2. Research Methods

The DBN model is a multi-layer neural network model structure formed by many RBM training modules stacked under certain requirements. The training of an artificial neuron network (ANN) is simultaneously conducted on all layers in a traditional manner, leading to a very high complexity of time. A DBN uses layer-by-layer training RBM to complete the training of the entire ANN, which effectively avoids the aforementioned problems. When the network depth is too deep, non-convex optimization problems readily appear. The DBN solution to this problem is to adopt a pre-training mechanism. Pre-training is a non-supervised process. The RBM is a bottom-up pre-trained layer-by-layer and the pre-training process replaces the operation of random initialization weight. After training, the network weights are tuned. A DBN has been successfully applied in many areas such as image recognition, speech recognition, and so on.

**Figure 2:**
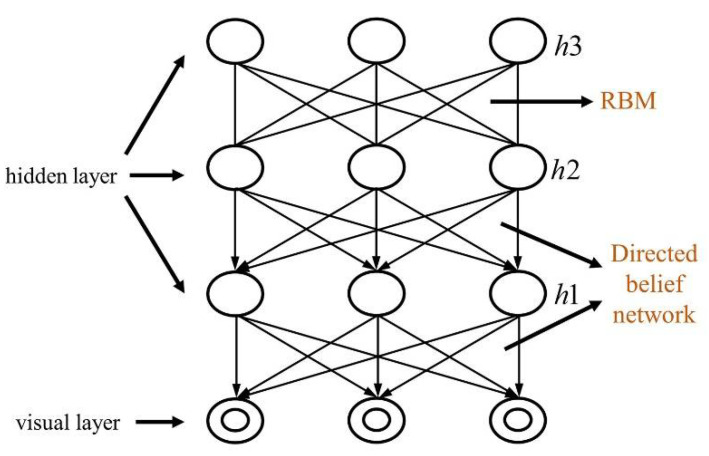
Deep belief network (DBN).

A DBN is a probabilistic generation model, thus it can be used both to identify and generate data. The DBN model establishes a joint distribution between input values and class labels; thus, it evaluates both generation and discrimination and both P (Observation | Label) and P (Label | Observation) processes. For the general discriminant model, there is only the latter, namely the P (Label|Observation) process. A DBN is composed of several RBM layers. Figure 2 shows a DBN model. The model includes a visual layer and hidden layer. The hidden layer is composed of three layers of neural networks. There are connections between adjacent layers but there is no connection between the cross-layer and same-layer nodes.

A back-propagation algorithm is the most commonly used method for neural network training. The back-propagation algorithm trains the entire network via iteration. The initialization of network parameters is also random. The back-propagation algorithm adjusts the parameters of the entire network by calculating the error between the top output value of the neural network and original input value. An update of each layer parameter is achieved via a gradient descent. Back propagation has many shortcomings that are mainly manifested as the method of randomly initializing network parameters and weakening the signal of a neural network, which may lead to the network reverting to a local optimal solution. To avoid these problems, a DBN adopts two mechanisms: pre-training and level-by-layer training. Pre-training is a bottom-up unsupervised training process in which the parameters of the neural network are initialized. Hinton’s layer-by-layer training is a type of deep network training and learning mechanism. It refers to the layer-by-layer training of a deep network, which is shown as a layer-by-layer training of the RBM in a DBN. The former initializes the parameters of the neural network through unsupervised training, which is more conducive to neural network learning and can successfully avoid the neural network reverting to a local optimum. The latter solves the problem of high complexity caused by the entire training of the neural network and accelerates the neural network convergence speed.

#### 2.2.1. DBN Model

**Figure 3:**
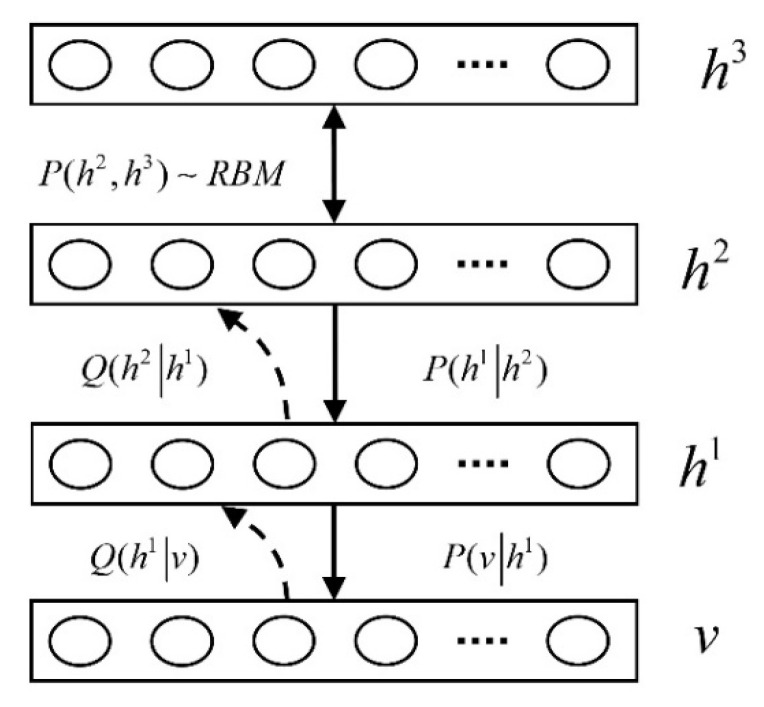
DBN model.

Figure 3 shows a basic DBN model. Formula (1) represents the joint distribution relationship between the visual layer V and the hidden layer as follows:

(1) $$P\left(v,h^{1},\ldots ,h^{l}\right)=\left(\overset{l-2}{\underset{k=0}{\prod }}P\left(h^{k}|h^{k+1}\right)\right)P\left(h^{l-1},h^{l}\right)$$

The visual layer is the conditional distribution of the visual layer value obtained from a given hidden layer, which is the joint distribution of the top layer. For the top-level classification task, the RBM classifies the abstract features extracted by hidden units using the SoftMax linear regression method. As shown in Figure 3, posterior probability Q is equivalent to the conditional distribution and top-level joint distribution to form a generating DBN model. At the K level, the probability that hidden units are activated can be determined using Formula (2) as follows:

(2) $$P\left(h_{i}^{k}=1|h^{k+1}\right)=\sigma \left(b_{i}^{k}+\underset{j}{\sum }W_{ij}^{k}h_{j}^{k+1}\right)$$

where $h_{i}^{k}$ is the value of the implicit unit i of the kth layer, $b_{i}^{k}$ is the weight of the kth layer implicit unit i offset, $W_{ij}^{k}$ is the hidden unit i, j of the kth layer to the k+1th layer.

#### 2.2.2. Layer-by-Layer Greedy Learning Algorithm

A layer-by-layer greedy learning algorithm was proposed by Hinton et al. in 2006 as a training method for deep neural networks. A DBN uses this algorithm to train neural networks. The training process is as follows:

(1) Initialize the parameter θ (B, C, W) in the K level model;

(2) Calculate the posterior probability value of the hidden unit j of the kth layer and randomly obtain the implicit unit value;

(3) According to the implicit unit value obtained during step (2), the visual unit value is reconstructed, P is calculated, and the implicit unit value is reconstructed by random sampling of P;

(4) According to the reconstructed visual unit, the hidden unit is calculated, P is calculated, and all hidden units are obtained by random sampling;

(5) Adjust the parameter θ (B, C, W) according to the rules of Equations (3)–(5) as follows:

(3) $$W^{k}\leftarrow W^{k}+\varepsilon \left(P\left(h_{1k}=1|v_{1k}\right)v_{1k}^{T}-P\left(h_{2k}=1|v_{2k}\right)v_{2k}^{T}\right)$$

(4) $$b^{k}\leftarrow b^{k}+\varepsilon \left(v_{1k}-v_{2k}\right)$$

(5) $$c^{k}\leftarrow c^{k}+\varepsilon \left(P\left(h_{1k}=1|v_{1k}\right)-P\left(h_{2k}=1|v_{2k}\right)\right)$$

(6) Repeat steps (1)–(5) until the termination condition is met and fix the layer parameters; and

(7) Repeat steps (1)–(6) to calculate the model parameters θ (b, c, W) for each layer of the RBM.

The starting point of the layer-by-layer greedy learning algorithm is the initial solution of the problem. Then, step-by-step, it is used to approximate the given target seeking a fast and sound solution. This idea has allowed for a new direction for solving the optimization problem related to the deep neural network structure.

#### 2.2.3. DBN Learning Process

During the process of learning the input data structure, DBN training is divided into two parts. The first part is pre-training, that is, unsupervised learning from bottom to top. The second part is the process of adjusting weight parameters, that is, top-down supervised learning. The specific processes are as follows:

(1) Unsupervised learning from the bottom up:

This process uses the sample data without a class label to train the parameters of each layer layer-by-layer. This step is the process of unsupervised training, which is the greatest difference between in-depth learning and a traditional neural network. Specifically, the first layer is trained with the unlabeled sample data to obtain the parameters of the first layer, and then the output of this layer is used as the input of the next layer, and so on, until the last layer is trained so that the parameters of each layer can be obtained.

(2) Top-down supervised learning:

This step is exactly the opposite of the first step. The parameters of the entire multi-layer model are adjusted according to the parameters of each layer obtained during the first step of training. This step is the process of supervising the training. The training of the first step can be regarded as the initialization process, thus it is termed the pre-training process, but this initialization is not random (otherwise it is meaningless) and is learned according to the sample data of the input unclassified label. Therefore, this initial value is nearer the global optimum than the value of the random initialization such that it can more quickly converge in subsequent training. Therefore, the effect of deep learning has a close relationship with the feature learning of the first step; of course, this is the advantage of deep learning.

## 3. Algorithm Design

By adjusting the experimental parameters, such as the number of neurons in different hidden layers, learning rate, number of iterations, and number of samples, the overall accuracy, confusion matrix, and kappa coefficient under different conditions are obtained and the results are analyzed.

### 3.1. Parameter Analysis

Analysis of the classification effect is an indispensable part of a classification experiment and evaluation standards must be established before comparing classification effects. The overall accuracy, confusion matrix, and kappa coefficient are commonly used to analyze classification results. The experiments in this study evaluated the effect of vegetation identification by using these three standards.

(1) Overall accuracy (OA) is the basic standard and the overall evaluation index of the classification results. The OA directly determines the feasibility of a classification method. If the classification accuracy of a classification method is too low, it has lost its practical value.

OA is equal to the total number of pixels divided by the number of pixels that are correctly classified as follows:

(6) $$p_{c}=\overset{m}{\underset{i=1}{\sum }}\frac{n_{ii}}{N}$$

where *p_c_* is the OA, *n_ii_* is the number of samples that are correctly identified in class I, *m* is the number of categories, and *N* is the total number of samples.

(2) The confusion matrix is used to compare information, such as the ratio of different types of outputs to real data, and it can characterize the accuracy of the classification’s output information in a matrix. The confusion matrix mainly calculates the coordinates and compares the two-dimensional coordinates of the image units measured on the spot with the corresponding image coordinates. The column elements in the matrix indicate the prediction category and the total number of each column represents the data that are predicted for that category. Each row represents the true class of the data and the total number of data in each row represents the number of data instances of that class. The confusion matrix reflects the classification results and the information of the real surface category, which is the basis of the OA and kappa coefficient. The formula for the percentages of each category’s classification result is as follows:

(7) $$p_{ij}=\frac{n_{ij}}{N}$$

where *n_ij_* is the number of samples belonging to class j predicted to be class I and *N* is the total number of samples. When *i* = *j*, it represents the classification accuracy of class I and $\overset{m}{\underset{i=1}{\sum }}\overset{m}{\underset{j=1}{\sum }}p_{ij}=1$.

(3)Kappa Coefficient. Firstly, the product of the total number of pixels N and the sum of the diagonal elements of the confusion matrix is calculated and then the sum of the total number of true pixels of a certain type of surface and the product of the total number of pixels that are mistaken for the category are subtracted. Next, the square of the total number of pixels is calculated and the sum of the total number of true pixels of a certain type of surface and the product of the total number of pixels that are mistaken for the category are subtracted. The ratio of these two is the kappa coefficient as follows:

(8) $$k=\frac{N\sum_{i=1}^{m}n_{ii}-\sum_{i=1}^{m}\left(n_{i*}n_{*i}\right)}{N^{2}-\sum_{i=1}^{m}\left(n_{i*}n_{*i}\right)}$$

where n_i*_ is the sum of *i*-th column data in the confusion matrix and n_*i_ is the sum of *j*-th row data in the confusion matrix.

The classification accuracy is defined by kappa coefficient as shown in Table 1.

### 3.2. Parameter Setting of DBN

This parameter setting adopts the control variable method. In the process of sample training and parameter value setting, the four parameter values of the five parameters remain unchanged, and the optimal value of the other index is obtained through sample training, and then the optimal value of the five parameters is obtained, and these five optimal values are the final parameter setting values.

(1) Number of network layers:

Hecht-Nielse clarified that there is a single information hidden layer for an ANN and a geometrically connected multi-layer biological network can achieve the desired geometric image projection effect. To decrease the discrimination bias and increase the accuracy, the channel layer can be added to the reserved value. However, the degree of program operation increases and the preprocessing requires more time. During this experiment, the different layers of a neural network were set up to compare the classification effect.

(2) Number of neurons in the hidden layer:

It is important to select the number of neurons in the hidden layer. If the amount of neurons in the hidden layer is too few, the training level of the neural network is not sufficient. If there are too many neurons, the accuracy will be improved in theory, but the preprocessing will take more time. Thus, the number of neurons should be carefully and properly selected. In this study, several numbers from 10 to 120 were selected as the number of hidden layer units to compare the classification accuracy, kappa coefficient, and training time, respectively. Then, the optimal value of the hidden layer units was obtained through analysis.

(3) Learning rate:

The learning rate determines the change in the classifier’s weight every time during the training process. A lower learning rate will cause the system to train at a low speed and a long convergence time. If the learning rate is set too high, the system will become unstable. According to the aforementioned analysis, during the experiment the learning rate was set as different values and the influence of learning rate on the recognition effect was observed.

(4) Number of iterations:

According to Hinton’s research, the batch size was set to 100. As the classification accuracy tends to be stable after 20 iterations of the sample, and the impact of a certain number of iterations on the classification results may be different for different problems, a different number of iterations was set during the experiment to observe the optimal value for vegetation recognition.

(5) Number of samples:

The number of samples also plays an extremely important role in the training of a network model. The greater the number of samples, the more comprehensive the characteristics of coverage. However, as the number of samples increases, the network training requires more time. Therefore, the number of training samples is not as high as possible. In the case of the same feature quantity, the smaller the number of samples, the more advantageous.

Therefore, the following experiments were mainly used to study the application of a DBN in vegetation remote sensing recognition by setting different network layers, number of neurons in each layer, learning rate, number of iterations, and number of samples. The control variable method is adopted to compare the influence of different parameter settings on classification results.

## 4. Experimental Parameters

The experiment was mainly conducted by setting different parameters for the DBN to explore the effect of the model on mine vegetation recognition. Satellite remote sensing images of vegetation-covered areas in the study area were selected, a size of 2000*1000 pixels, and input into the model for sample training. These data are based on pixels, a total of 2000,000 pixels. The selected elements of this identification training are mainly color and satellite image color saturation. The vegetation-covered area is green and the non-vegetation-covered area is yellowish-brown. The parameters to be learned during this experiment included the depth of the network, node number in the hidden layer, learning rate, number of iterations, and training sample size. The classification results were evaluated from three aspects, including OA, confusion matrix, and kappa coefficient. The details are as follows:

### 4.1. Number of Network Layers

**Figure 4:**
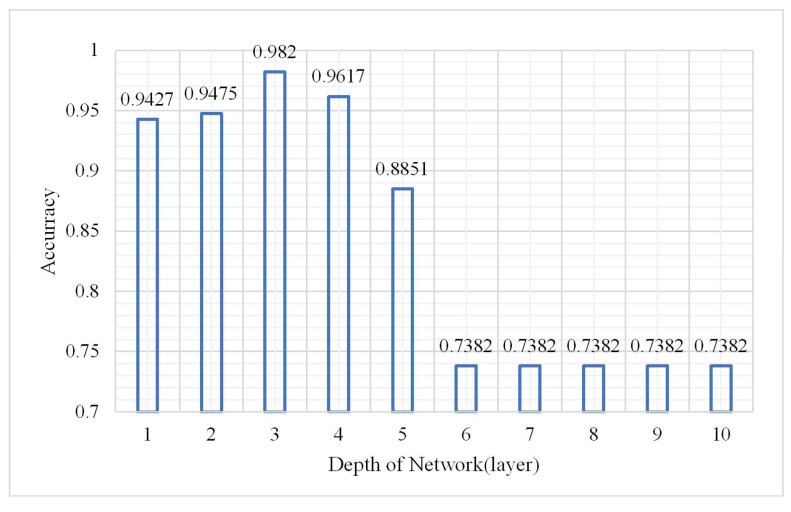
Change in overall accuracy.

**Figure 5:**
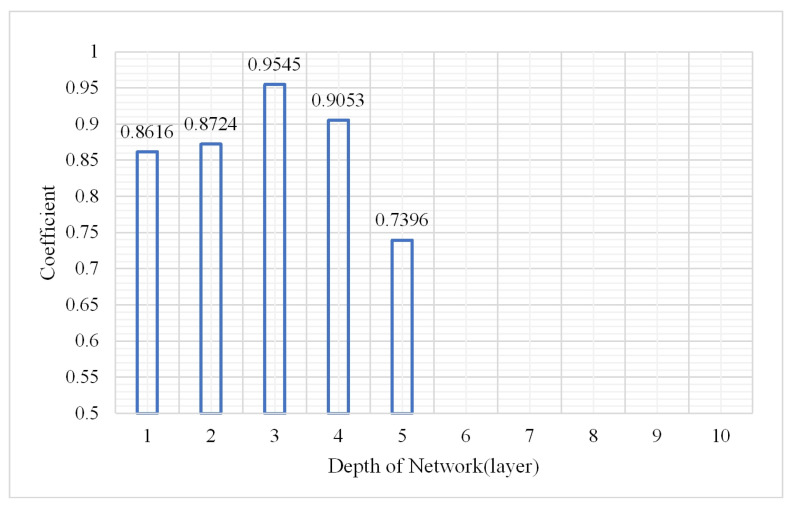
Change in kappa coefficient.

**Figure 6:**
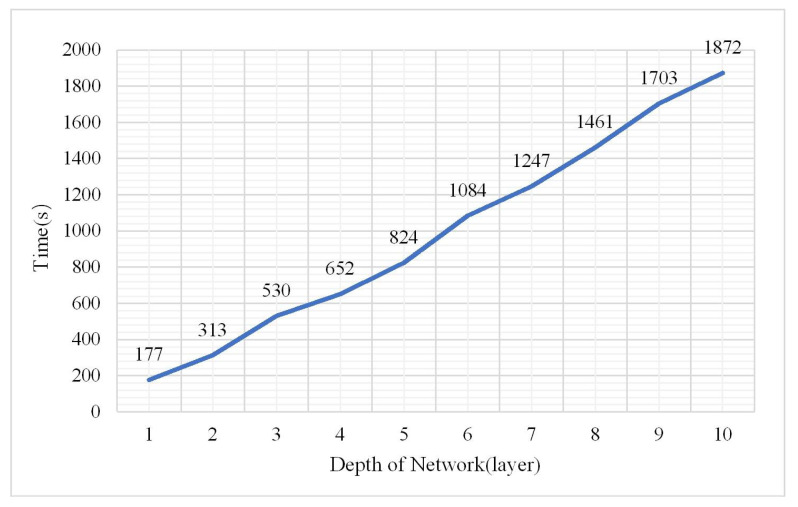
Change in training time.

As a deep learning model, the depth of the DBN model is certainly a main factor affecting the classification result. More abstract features can be extracted with deep layers; however, the model may be overfitted under this condition. The experiment assessed the change in the OA, confusion matrix, and kappa coefficient under hidden layers from 1 to 10. The parameters were as follows: the node of a hidden layer was 50, the learning rate was 0.01, the number of iterations was 3, and the sample size was 2,000,000. The results are shown in Figure 4, Figure 5 and Figure 6.

**Figure 7:**
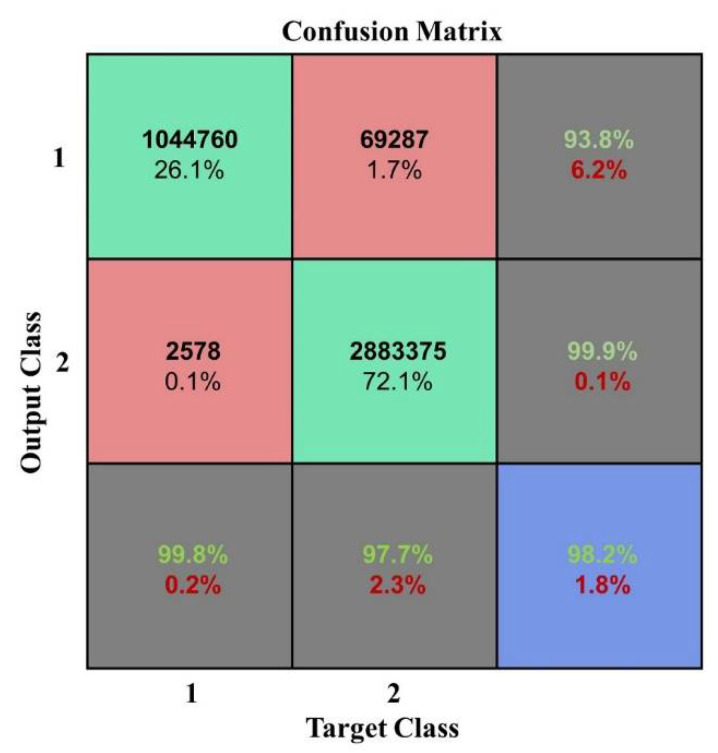
Confusion matrix at a network depth of three.

As show in Figure 4 and Figure 5, the accuracy is significantly better when the numbers of layers were from 1 to 5 which indicated that the model performed well when the depth was 1–5. The best classification accuracy and kappa coefficient were 0.982 and 0.9545, respectively, at a depth of 3. In addition, when the hidden layer number exceeded 5, the classification accuracy remained unchanged at 0.7382 while the kappa coefficient was 0. Thus, the positive sample (vegetation) accounts for 73.82% of the total samples in the test data while the DBN after training identified all the test samples as vegetation. The reason for this classification error is that the hidden layer number is too large such that the model is over fitted. Thus, the classification result is not ideal. The confusion matrix when the network depth was three is shown in Figure 7.

### 4.2. Number of Neurons in the Hidden Layer

**Figure 8:**
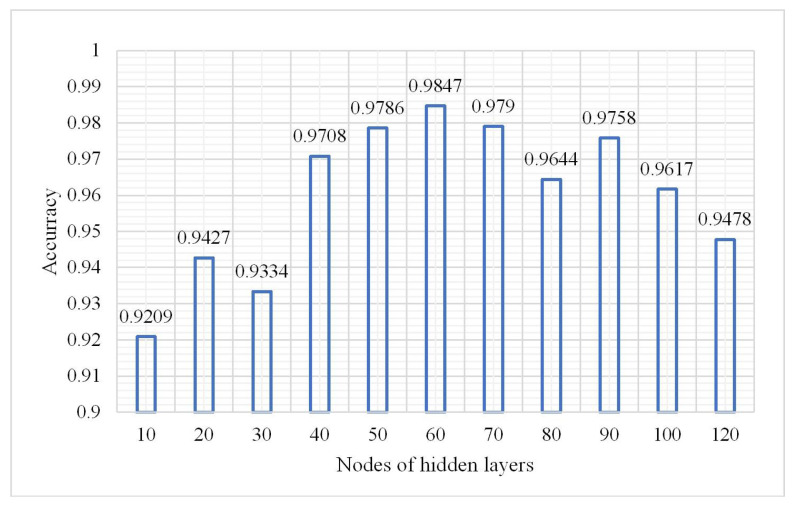
Change in overall accuracy.

**Figure 9:**
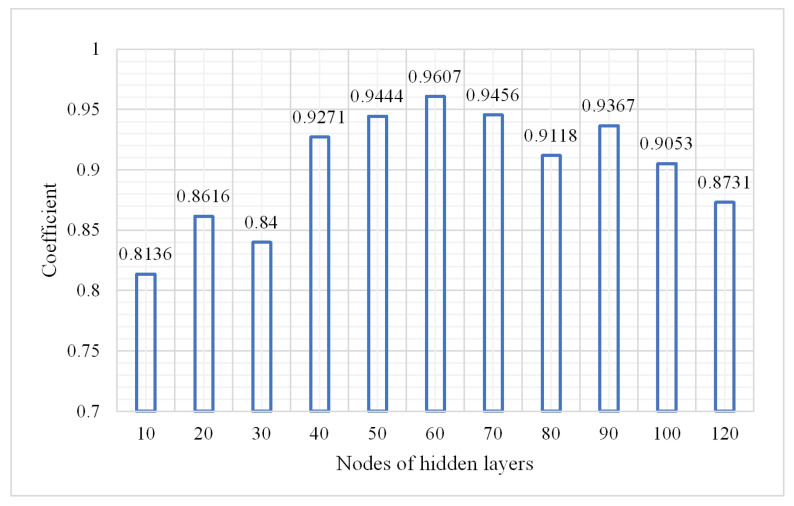
Change in kappa coefficient.

**Figure 10:**
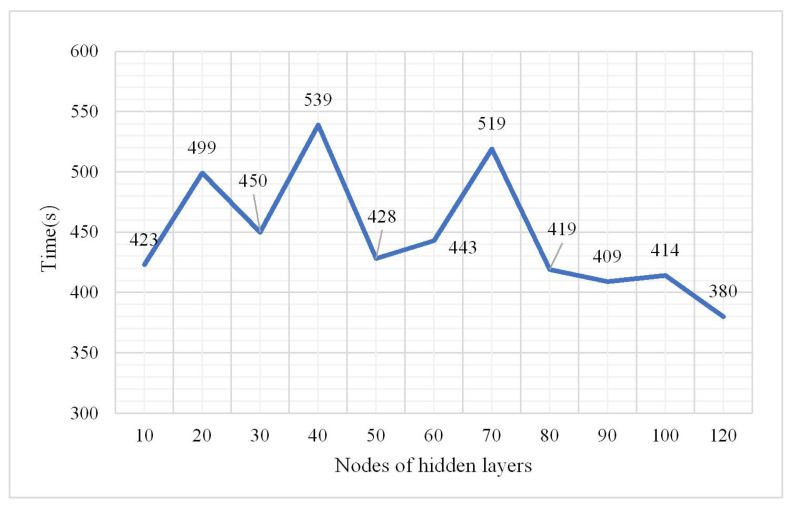
Change in training time.

From (1), the overall classification result is the best when the number of hidden layers is 3. In the following experiment the hidden layers were fixed at three to explore the change in the OA, confusion matrix, and kappa coefficient. The parameters were as follows: a learning rate of 0.01, number of iterations of 3, and a sample size of 2,000,000. The results are shown in Figure 8, Figure 9 and Figure 10.

**Figure 11:**
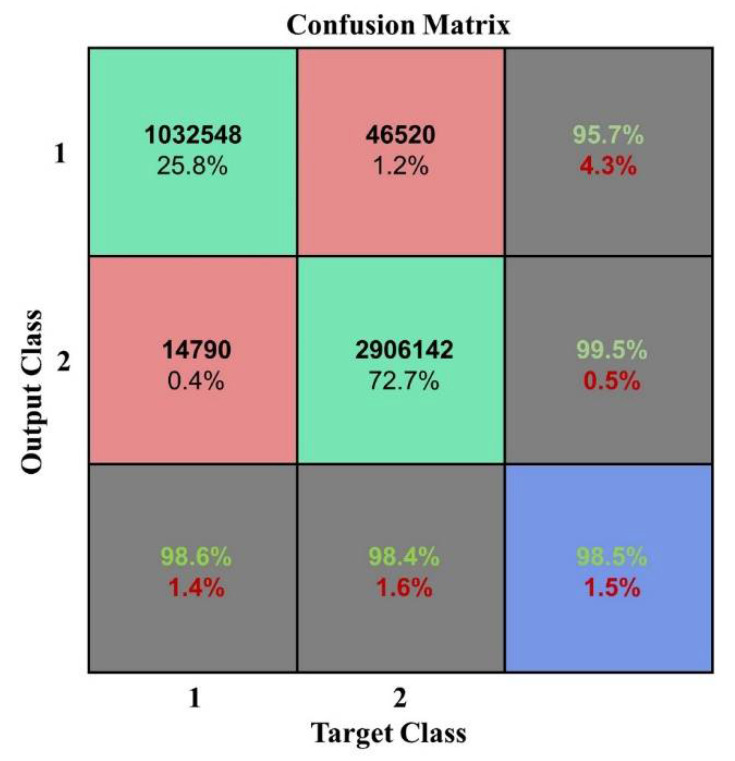
Confusion matrix when the nodes of the hidden layers were 60.

The model performed best when the number of nodes of the hidden layers was set 60 according to Figure 8 and Figure 9. So in the following experiment, the number was maintained at 60. The confusion matrix is shown in Figure 11.

### 4.3. Learning Rate

**Figure 12:**
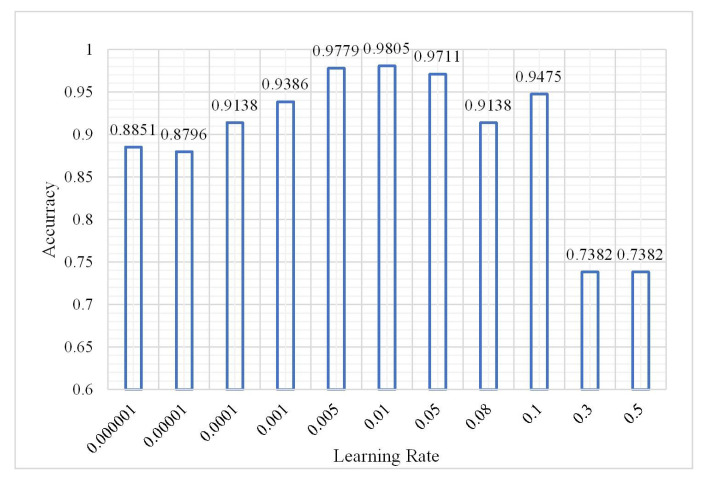
Change in overall accuracy.

**Figure 13:**
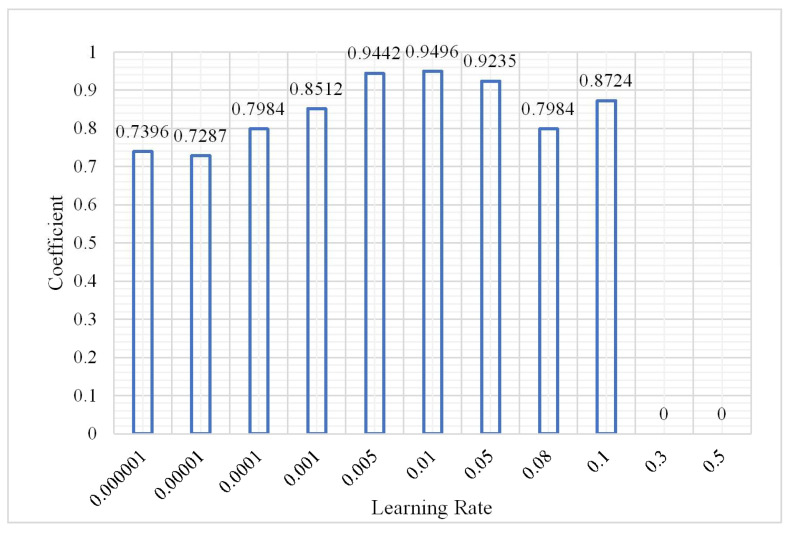
Change in kappa coefficient.

**Figure 14:**
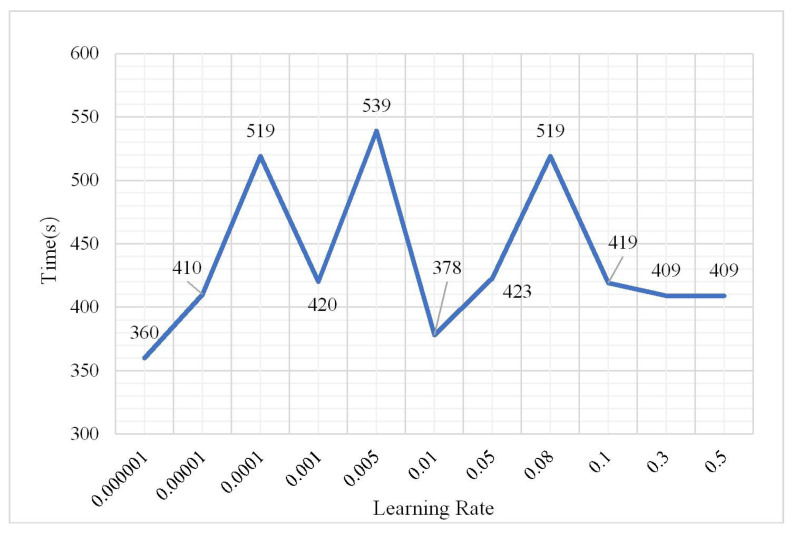
Change in training time.

The DBN model could obtain the best classification result when the number of hidden layers was 3 and the number of nodes of the hidden layers was 60 according to (1) and (2). Without changing the parameters, the number of iterations was 3 and the sample size was 2,000,000. The classification result was analyzed as the learning rate changed from 0.000001 to 0.5. The results are shown in Figure 12, Figure 13 and Figure 14.

**Figure 15:**
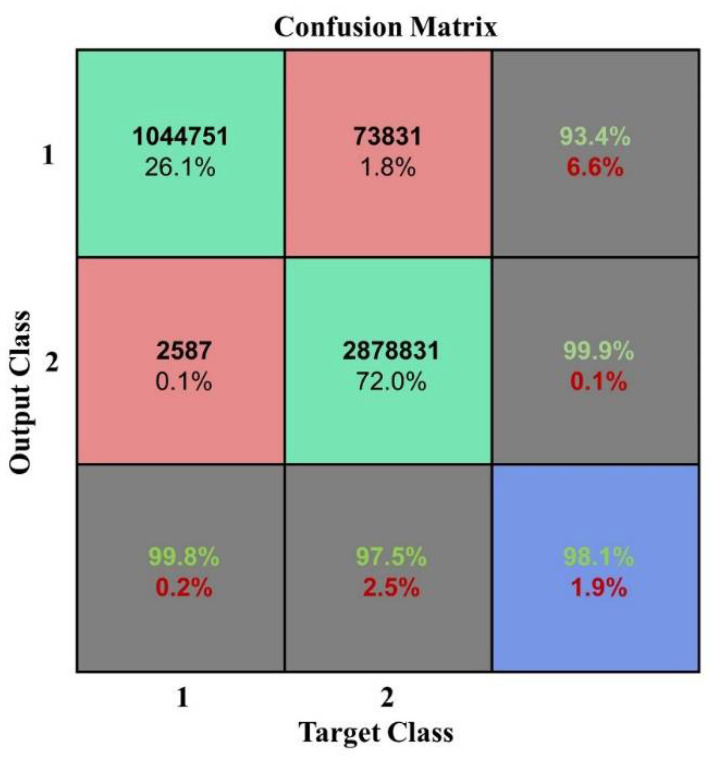
Confusion matrix at a learning rate of 0.01.

As shown in the aforementioned figures, the classification OA shows a gradual upward trend as the learning rate changes from 0.000001 to 0.1. However, when the learning rate is greater than 0.3, the OA decreases to 0.7382. The overfitting phenomenon occurs in this situation. However, the training time fluctuates up and down before overfitting and there is no obvious upward or downward trend; however, in the case of overfitting, the training time shows a decreasing trend with the increase in learning rate. It can be concluded that when the learning rate of the neural network is slower, it is better. Properly adjusting the value of the learning rate can obtain better classification results. To obtain a more accurate classification result without consuming considerable training time, during the following experiments the learning rate was set to 0.01. The confusion matrix shown in Figure 15 has a learning rate of 0.01.

### 4.4. Number of Iterations

**Figure 16:**
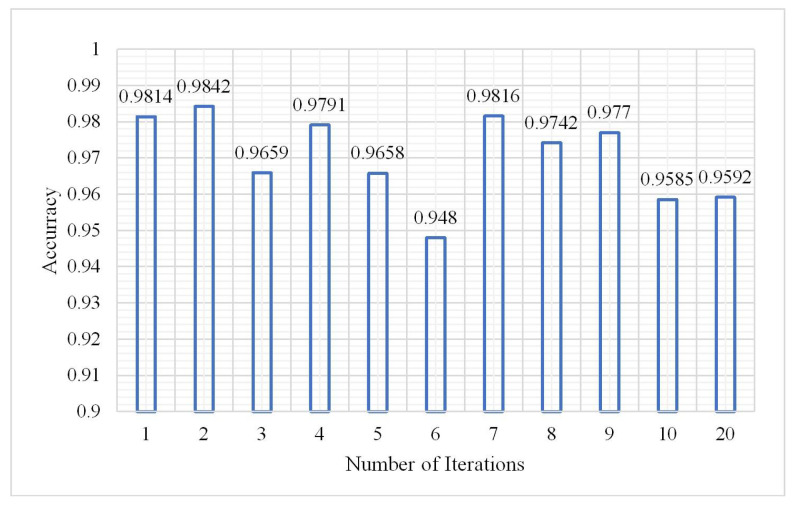
Change in overall accuracy.

**Figure 17:**
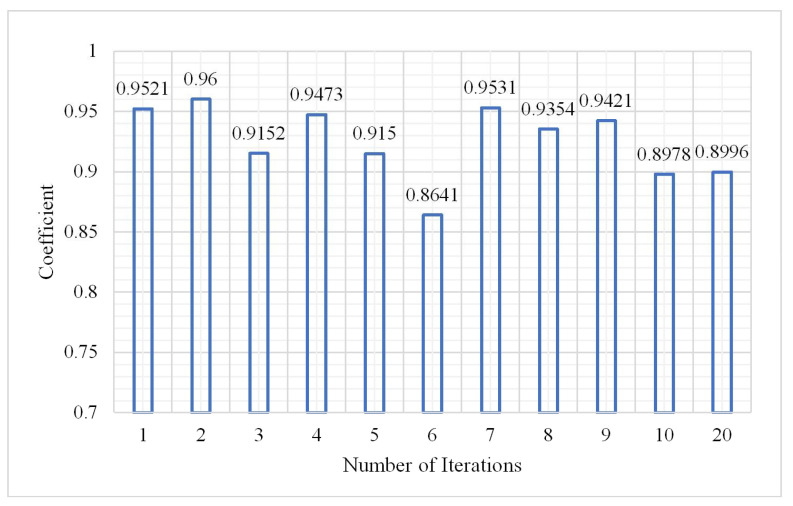
Change in kappa coefficient.

**Figure 18:**
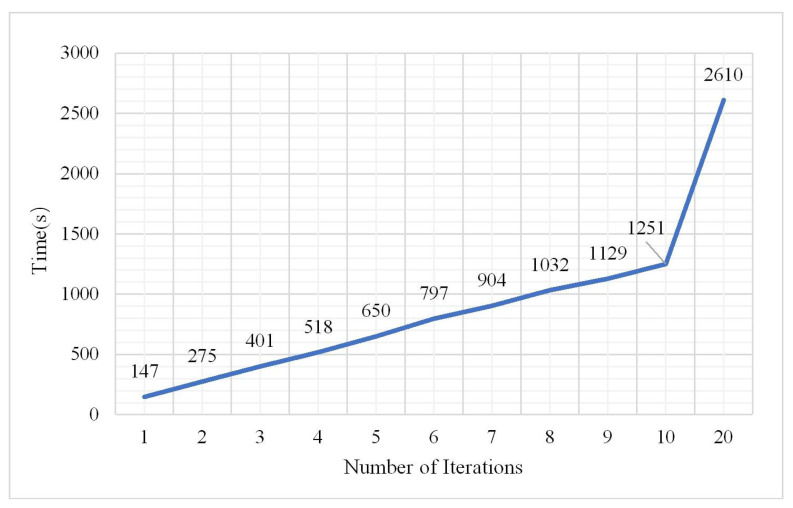
Change in training time.

The number of iterations has a great influence on the classification result. The number of iterations in the experiments was set from 1 to 20. The parameters from the analysis of (1), (2), and (3) showed that when the number of hidden layers was 3, the number of neurons in each hidden layer was 60, and the learning rate was 0.01, and the classification result was the best. Maintaining the sample size at 2,000,000, the classification results are shown in Figure 16, Figure 17 and Figure 18.

**Figure 19:**
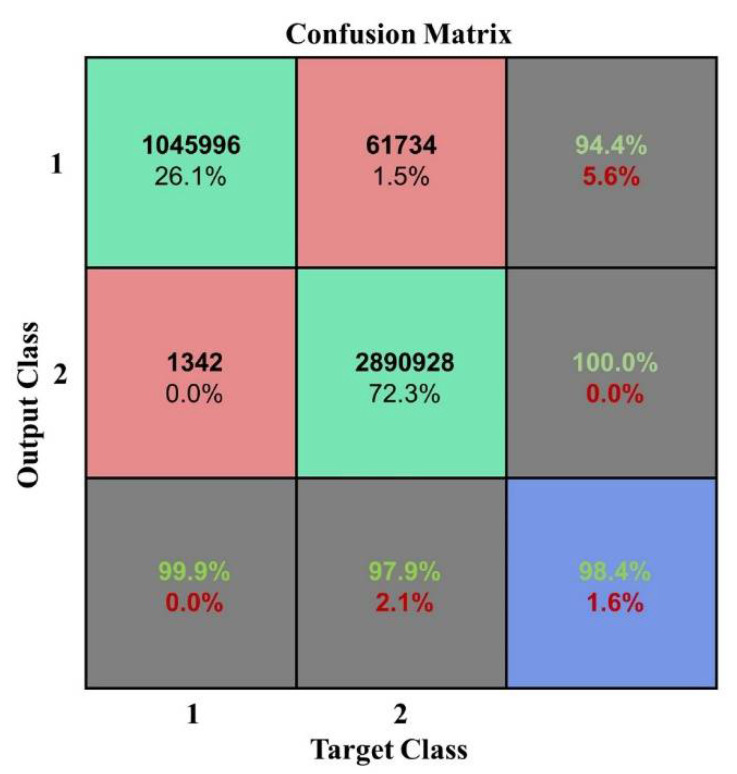
Confusion matrix at a number of iterations of 2.

From the histogram of the change in the OA and kappa coefficient with the number of iterations, it can be seen that different iterations will lead to some differences in classification results under the same conditions; however, the OA and kappa coefficient do not show an increasing or decreasing trend while the training time significantly increased as the number of iterations increased. During the experiment, the maximum number of iterations was 20, but the OA and kappa coefficient did not significantly change. When the number of iterations was 2, the classification effect was obvious and the training time was not too long. The confusion matrix is shown in Figure 19 when the number of iterations was 2.

### 4.5. Sample Size

**Figure 20:**
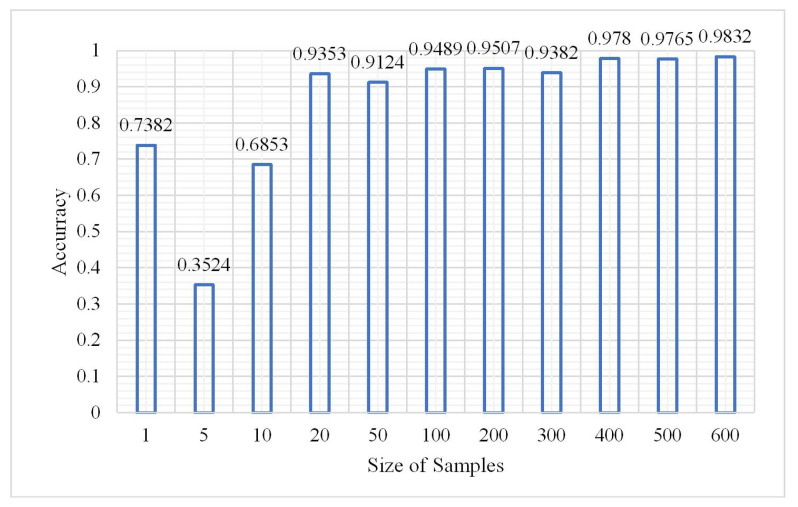
Change in overall accuracy.

**Figure 21:**
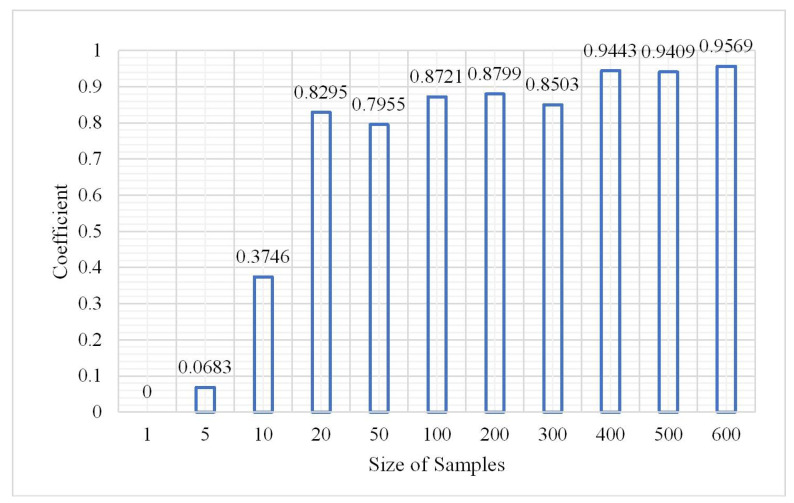
Change in kappa coefficient.

**Figure 22:**
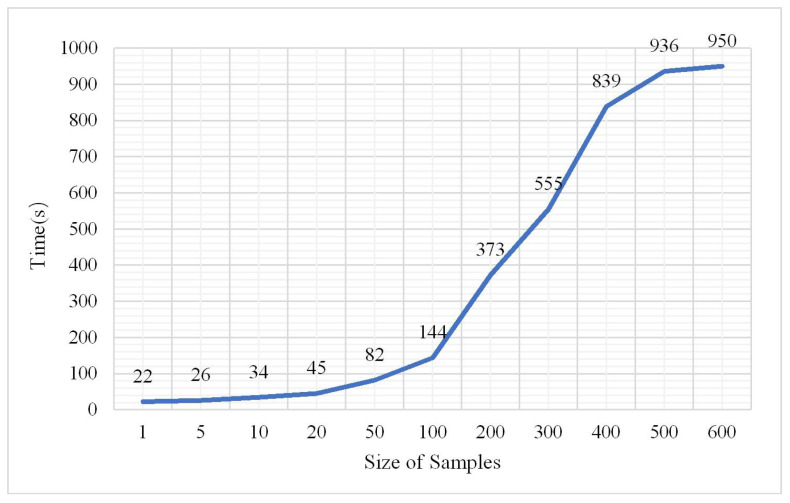
Change in training time.

In terms of probability, more samples reflect more comprehensive features, and the learning network can also more accurately identify ground objects. However, too many samples will inevitably consume considerable time for training data. The number of samples during the experiment was from 10,000 to 6,000,000 and the other parameters were measured via the aforementioned experiments. The classification results are shown in Figure 20, Figure 21 and Figure 22.

**Figure 23:**
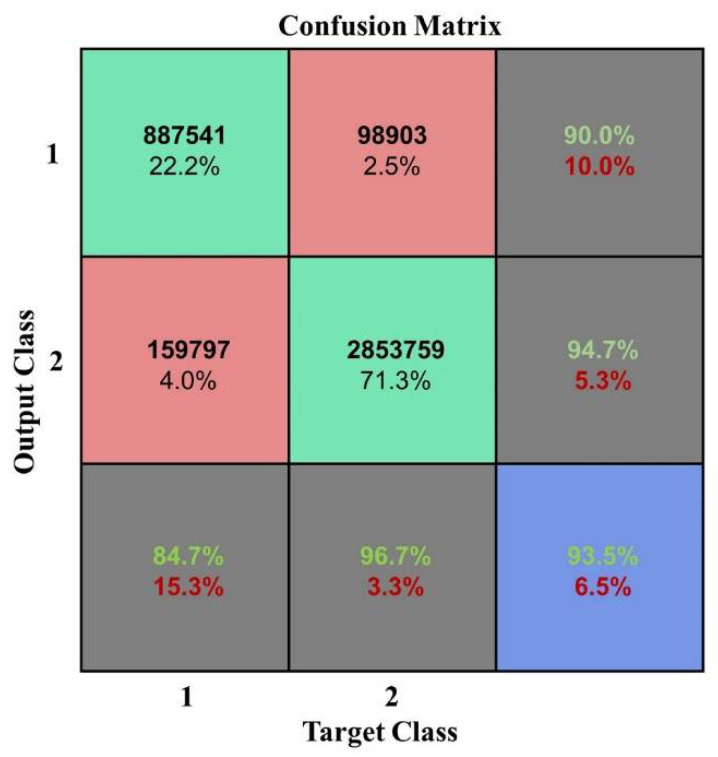
Confusion matrix at a sample size of 2,000,000.

According to the OA and kappa coefficient histogram, when the number of samples is less than 200,000, the OA is less than 0.8. When the number of samples is greater than 200,000, the OA is higher and tends to be stable above 0.9, the kappa coefficients are nearly all greater than 0.8, and the training time significantly increases with the increase in sample size. The confusion matrix at a sample size of 2,000,000 is shown in Figure 23.

## 5. Experimental Results and Discussion

According to the above experimental design, parameter settings: Number of network layers is 3, number of neurons in the hidden layer is 60, learning rate is 0.01, number of iterations is 2, and the sample size is 2,000,000. The optimal experimental result is obtained, the optimal parameters for the DBN recognizing the vegetation of the mine are listed in Table 2.

**Figure 24:**
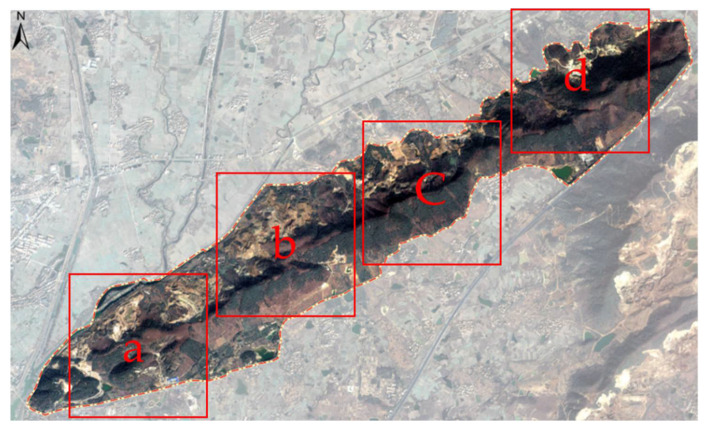
Recognition areas. (**a**) Satellite image slice named recognition area “a”. (**b**) Satellite image slice named recognition area “b”. (**c**) Satellite image slice named recognition area “c”. (**d**) Satellite image slice named recognition area “d”.

Set the optimal parameter values, and select four recognition areas a, b, c, and d in the research area (as shown in Figure 24), and use the DBN algorithm to identify vegetation.

**Figure 25:**
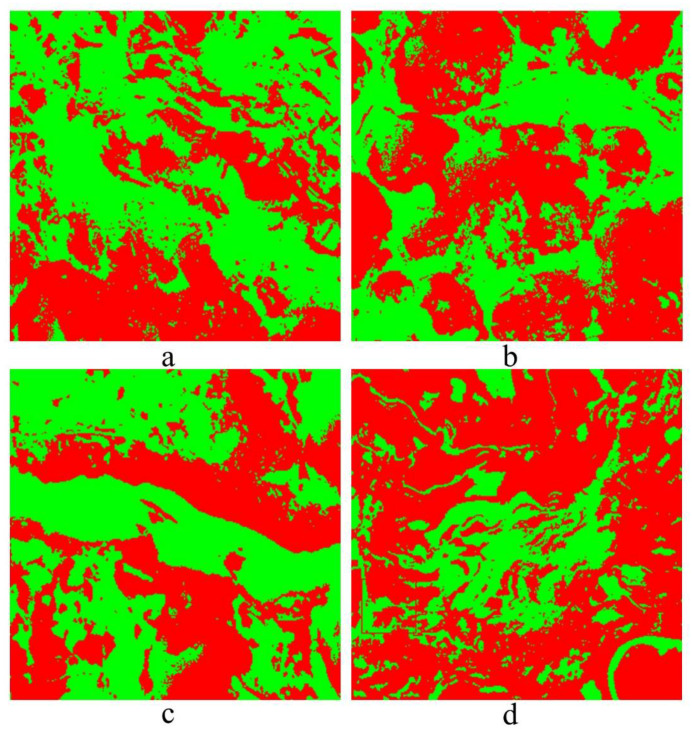
Areas **a**–**d**.

Figure 25 shows the performance of recognition for four different areas which were cropped from the original remote sensing image, the red represents the vegetation covered area, while the green represents the area without vegetation covered.

**Figure 26:**
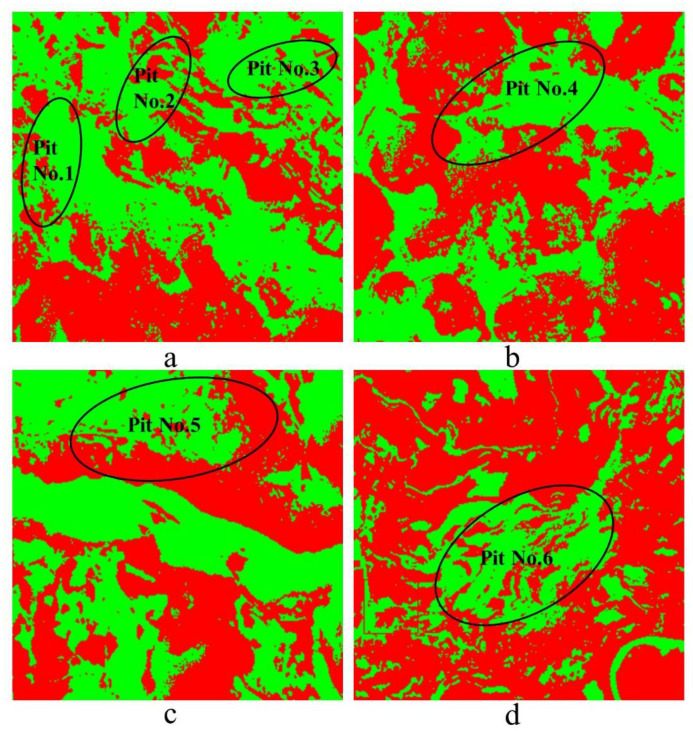
Pit on the areas **a**–**d**.

The four recognition areas of a, b, c, d of six mine position is shown in Figure 26. By identifying the results, it can be seen that the most obvious results are in the coal mine, d area of Pit No.6. The b and c area of the Pit No.4 and No.5 mine areas with and without contrast significant effect of vegetation area, vegetation of the Pit No.1, 2, 3 area, and the surrounding without vegetation area are identified, and the vegetation coverage area of the difference is obvious.

Through comparison and demonstration, it is concluded that with the setting of the optimal parameter value, the vegetation-covered area and non-vegetation-covered area can be effectively identified, which is basically consistent with the remote sensing image and the actual situation, and can more intuitively reflect the vegetation coverage area scope in the site.

The OA, kappa coefficient, and confusion matrix of the recognition results for the four areas are respectively shown in Table 3 and Table 4.

It can be concluded from the aforementioned tables that the accuracy of the vegetation recognition for the four random areas trimmed from the remote sensing image are basically stable. The overall accuracy for each area is 98.5%, 97.5%, 93.5%, and 94.3% and the kappa coefficient is 0.98, 0.97, 0.93, and 0.93, respectively. The recognition results show that the higher the Kappa coefficient is, the better the recognition effect is. Experiments show that DBN technology can effectively identify the vegetation coverage of the site, with an average recognition rate of 95.95% and a high accuracy of kappa coefficient of 0.95. The average recognition rate of 95.95% outperforming some other models [19], indicates that the setting of the model has basically reached the optimal.

The experiments show the feasibility of applying the DBN to mine vegetation identification. A higher Kappa coefficient means a higher resolution picture, the clearer the satellite image is, the more accurate the recognition result is. The model can provide effective monitoring data for the mine ecological environment if used to continuously observe the overall mine vegetation coverage.

## 6. Conclusions

Experiments proved the feasibility of using deep learning in the application of remote sensing vegetation identification. When the sample size was 2,000,000 pixels, through repeated tests and classification effect comparison, the optimal parameter setting suitable for mine vegetation identification was obtained. Parameter setting: the number of network layers was 3 layers; the number of hidden layer neurons was 60, the learning rate was 0.01 and the number of iterations was 2. The average identification accuracy of DBN technology reached 95.95%, outperforming some other models [19], which can accurately reflect the vegetation coverage. The clearer the satellite image was, the more accurate the recognition result was, and the accuracy was closer to 100%. By determining the coverage of mine vegetation, the damage degree of mine vegetation and the area that needs ecological vegetation restoration could be determined, which provides a data basis for decision-making departments. At the same time, it can reduce the costly field survey work and save time. This identification technology is of great significance for mine ecological restoration and ecological vegetation restoration.

## Figures and Tables

**Table 1:** Relationship between the kappa coefficient and classification accuracy.

Classification Accuracy	Kappa Coefficient
Very poor	< 0
Poor	0.00–0.20
Average	0.20–0.40
Good	0.40–0.60
Very good	0.60–0.80
Excellent	0.80–1.00

**Table 2:** DBN parameters.

Parameter	Depth of the Network	Number of Nodes for the Hidden Layer	Learning Rate	Number of Iterations	Number of Samples
Value	3	60	0.01	2	2,000,000

**Table 3:** Area (a).

Categories	Vegetation				Other Objects				User Accuracy			
	Area (a)	Area (b)	Area (c)	Area (d)	Area (a)	Area (b)	Area (c)	Area (d)	Area (a)	Area (b)	Area (c)	Area (d)
Vegetation	25.8%	25.0%	26.0%	26.2%	1.2%	1.0%	6.5%	5.7%	95.7%	96.3%	80.1%	82.1%
Other Objects	0.4%	1.2%	0.2%	0.0%	72.7%	72.8%	67.3%	68.1%	99.5%	98.4%	99.7%	100%
Production Accuracy	98.6%	95.5%	99.3%	100%	98.4%	98.7%	91.2%	92.2%	98.5%	97.9%	93.3%	94.3%

**Table 4:** Overall accuracy and Kappa coefficient.

Categories	Area (a)	Area (b)	Area (c)	Area (d)
Overall accuracy	98.5%	97.9%	93.5%	94.3%
kappa coefficient	0.98	0.97	0.93	0.93
